# Oral Manifestations of Multiple Sclerosis: A Systematic Review

**DOI:** 10.3390/jcm14092944

**Published:** 2025-04-24

**Authors:** Paula García-Rios, Francisco Javier Rodríguez-Lozano, Miguel Ramón Pecci-Lloret

**Affiliations:** Gerodontologý and Special Care Dentistry Unit, Faculty of Medicine, Morales Meseguer Hospital, University of Murcia, IMIB-Arrixaca, 30008 Murcia, Spain; paula.garciar@um.es (P.G.-R.); miguelramon.pecci@um.es (M.R.P.-L.)

**Keywords:** multiple sclerosis, oral manifestations, oral ulcers, periodontal diseases, systematic review, xerostomia

## Abstract

**Background:** Multiple sclerosis (MS) is a chronic autoimmune disease of the central nervous system characterized by diverse clinical manifestations, including the potential involvement of the oral cavity. Oral symptoms in MS patients may arise both as direct consequences of the disease and as side effects of pharmacological treatments. These manifestations, such as xerostomia, periodontal disease, and dental sensitivity, can significantly affect quality of life and may be underrecognized in clinical practice. **Aim:** To systematically assess the presence and relevance of oral manifestations in patients with MS, and to identify correlations between these symptoms and clinical parameters such as MS phenotype, disease duration, and disability level. Materials and Methods: A systematic review was performed following Preferred Reporting Items for Systematic Reviews and Meta-Analyses (PRISMA) guidelines. A database search was conducted in PubMed and Scopus on 17 March 2025, using terms related to “multiple sclerosis” and “oral manifestations”. Inclusion criteria were limited to observational studies published in the last ten years, focusing on oral symptoms in MS patients. Furthermore, the quality of the studies was assessed following the Newcastle–Ottawa Scale (NOS) for cohort and case–control studies, and the JBI Critical Appraisal checklist for analytical cross-sectional studies. **Results:** Ten studies met the inclusion criteria. The most frequently reported oral manifestations were hyposalivation, gingival inflammation, increased DMFT and plaque indices, dental sensitivity, and oral pain. Several studies found statistically significant associations between oral dryness and MS phenotype (*p* < 0.05), and between periodontal health and degree of disability (*p* < 0.05). However, heterogeneity in methodology and lack of longitudinal studies were noted as limitations. **Conclusions:** This review highlights a clear relationship between MS and several oral health disturbances, particularly xerostomia and periodontal disease. The findings underscore the need for multidisciplinary care approaches and further studies with standardized protocols to better understand oral-systemic interactions in MS.

## 1. Introduction

Multiple sclerosis (MS) is a chronic, immune-mediated inflammatory disease of the central nervous system (CNS), characterized by demyelination, axonal injury, and neurodegeneration. Due to its capacity to affect any region of the CNS—including the brain, spinal cord, and optic nerves—it presents with a wide range of heterogeneous signs and symptoms, such as motor deficits, sensory disturbances, visual impairment, and cognitive dysfunction. MS is one of the leading causes of non-traumatic neurological disability in young adults, predominantly affecting individuals between the ages of 20 and 40, and with a higher incidence in women. A recent global meta-analysis published in 2023 estimated the worldwide prevalence of MS at approximately 35.9 per 100,000 population, with significant regional variability—ranging from 2.1 per 100,000 in sub-Saharan Africa to over 200 per 100,000 in parts of Europe and North America. These disparities are attributed to genetic, environmental, and socioeconomic factors, as well as diagnostic capacity. The burden of MS extends beyond physical impairment, as it carries profound psychosocial and economic consequences, including increased healthcare costs, loss of productivity, and decreased quality of life among young adults in their prime working years [1,2].

This chronic inflammatory disorder is mediated by activated T lymphocytes, although the involvement of B lymphocytes and cells of the innate immune system is becoming increasingly evident. Neuronal damage and axonal loss is caused by demyelination and subsequent degeneration of the CNS [3].

The clinical variability of multiple sclerosis (MS) lies in its diverse involvement of the central nervous system, affecting motor, sensory, visual, and autonomic pathways, and resulting in characteristic symptoms such as optic neuritis, Uhthoff’s phenomenon, and Lhermitte’s sign. Additionally, disabling features like fatigue, cognitive decline, bladder dysfunction, and spasticity are common. Although its etiology remains unclear, current evidence suggests an interaction between genetic predisposition and environmental factors—particularly low vitamin D levels, Epstein–Barr virus infection, mononucleosis, and smoking—as key contributors to disease onset [4,5,6,7,8].

On the other hand, genetic susceptibility also influences the development of the disease. First-degree relatives of MS patients have a higher risk of MS compared to the general population, and it has been shown that the presence of the human leukocyte antigen (HLA) class II haplotype HLA-DR2 region on chromosome 6p21 is significantly associated with MS [9,10].

The diagnosis of MS is diverse and complex. The clinic remains a fundamental pillar to define the disease, based on a complete anamnesis and a neurological examination to establish the temporal and spatial dissemination of some symptoms and clinical signs [11]. On the other hand, paraclinical tests such as magnetic resonance imaging (MRI) or cerebrospinal fluid (CSF) analysis are also relevant. The former is based on the detection of CNS inflammatory lesions while the latter evaluates mismatched oligoclonal immunoglobulin bands in the CSF. In addition, it is worth mentioning the use of the McDonalds diagnostic criteria, which aim to make an early diagnosis in order to apply treatment earlier and more effectively [11,12]. The last aspect to consider in order to make a good diagnosis of the disease is the establishment of differential diagnoses with certain pathologies such as Devic’s syndrome [13]. The most common are clinically isolated syndrome, radiologically isolated syndrome, relapsing–remitting multiple sclerosis (RRMS), primary progressive multiple sclerosis (PPMS), and secondary progressive multiple sclerosis (SPMS) [14].

Orofacial signs of MS may be early manifestations or associated with disease progression. Among the most common are trigeminal neuralgia-type facial pain, paresthesias, hemifacial spasms, and mild dysarthria, which may present episodically and painfully. Motor disturbances such as facial tonic spasms, muscle weakness, and temporomandibular disorders affecting chewing and speech may also be observed [15,16]. These symptoms are often confused with dental pathologies, so it is key for the dental professional to recognize them in order to refer to the neurologist. In addition, patients may suffer from dry mouth and impaired oral hygiene due to physical limitations and adverse effects of treatments [17].

Because the clinical manifestations of this pathology are also evident at the oral level—either as a direct consequence of the disease or as a result of the treatment used—it is essential to identify the most common forms of presentation, given the bidirectional relationship between oral and systemic involvement. Therefore, summarizing studies on the oral manifestations of multiple sclerosis is of great importance.

The general objective of this systematic review is to provide a qualitative synthesis of existing studies addressing the oral manifestations of multiple sclerosis (MS). The specific objectives are as follows: to examine the ways in which multiple sclerosis affects the oral cavity; to identify the oral manifestations most frequently associated with the disease; to assess the significance of patients’ perception of these manifestations in relation to MS; and to explore the association between specific oral manifestations and particular population groups.

## 2. Materials and Methods

This systematic review was carried out in accordance with the updated PRISMA 2020 (Preferred Reporting Items for Systematic Reviews and Meta-Analyses) guidelines. Additionally, the review was registered in the PROSPERO database (International Prospective Registry of Systematic Reviews) with the registration number CRD420251022678, on 30 March 2025.

The following inclusion criteria were applied in this review: Articles published in the last 10 years, i.e., between 2015 and 2025 and those examining oral manifestations present in patients with multiple sclerosis were included in this review. Articles that matched our search terms were also accepted. In contrast, articles that only mentioned systemic manifestations of MS were rejected, as well as those that did not meet our inclusion criteria. In order to establish the inclusion criteria, these should follow the PICO model: population/problem (P): patient with multiple sclerosis; intervention (I): not applicable; comparison/control (C): healthy patients; outcomes (O): existing oral manifestations in MS patients. Based on the above, it can be determined that our PICO question is as follows: what are the oral manifestations in patients with multiple sclerosis?

### 2.1. Search Strategy

PubMed, Scopus, Scielo, and The Cochrane Library were the databases chosen for an exhaustive search of articles, with the aim of identifying and studying those that incorporated relevant information on the topic proposed for this review. The search was carried out on 17 March 2025. The terms used to carry out the search were those obtained from the Mesh (Medical Subject Heading) thesaurus. Those referring to the term multiple sclerosis are ‘multiple sclerosis’ OR ‘multiple sclerosis, relapsing-remitting’ OR ‘multiple sclerosis, progressive’ OR ‘multiple sclerosis, chronic progressive’ OR ‘multiple sclerosis, primary progressive’ OR ‘multiple sclerosis, secondary progressive’ OR ‘multiple sclerosis, clinical remission’ OR ‘demyelinating disease’; and those referring to the term oral manifestations are ‘oral manifestations’ OR “manifestation, oral” OR “manifestations, oral” OR “oral manifestation” OR “oral lesions” OR “oral ulcers” OR “mucosal lesions” OR “oral health”. Boolean operators (‘AND’ and ‘OR’) were used to relate the terms explained above to each other. The results obtained after searching for articles in the databases used are shown in the Table 1.

### 2.2. Study Selection

We started by discarding duplicate studies by entering the articles resulting from the search into the bibliographic manager Mendeley (Elsevier). Then, on the basis of title and abstract, and in compliance with the established inclusion and exclusion criteria, a first selection of articles was made. Finally, the remaining studies were read and analyzed in full text, thus corroborating their compliance with the predetermined objectives.

### 2.3. Data Extraction

For data extraction, the evaluators (PGR and FJRL) considered the following categories were taken into account in each of the articles studied: authorship and year of publication; type of study; most frequent oral manifestations; treatment; differential diagnosis. In the absence of an agreement, a third assessor (MRPL) analyzed the points of controversy and made a final decision with respect to predefined inclusion criteria and further analysis of the study.

### 2.4. Quality Analysis

This systematic review is composed of 5 case–control studies and 5 cross-sectional studies. To conduct the quality assessment of the first five, the Newcastle–Ottawa Scale (NOS) [18] was used, which serves to evaluate the methodological quality of case–control or cohort studies. The scale assigns “stars” based on the fulfillment of criteria across three domains: selection, comparability, and exposure in the case of case–control studies. Comparability can be awarded a maximum of two stars, while the remaining domains and their corresponding items can each receive a maximum of one star. Articles that scored between 7 and 9 stars were categorized as having a low risk of bias; those with scores between 4 and 6 stars were considered to have a moderate risk; and those with scores between 0 and 3 stars were classified as having a high risk of bias. On the other hand, the quality assessment of the cross-sectional studies was carried out using the Joanna Briggs Institute’s (JBI) Critical Appraisal tool [19], which defines what observational studies should include, thanks to the set of recommendations it provides. This appraisal tool consists of eight evaluation criteria that examine the methodological quality of an article and determine the extent to which a study has addressed potential biases in its design, conduct, and analysis. Each criterion was evaluated and marked as “yes”, “no”, “unclear”, or “not applicable”. Inclusion in the review required meeting at least four of the eight requirements, with a total score of at least 50%. The final scores assigned by each reviewer were checked for discrepancies, which were resolved by consensus. Two reviewers (PGR and FJRL) independently assessed the studies. In case of disagreement, a third reviewer (MRPL) was consulted to reach a consensus.

## 3. Results

### 3.1. Study Selection and Flow Diagram

**Figure 1:**
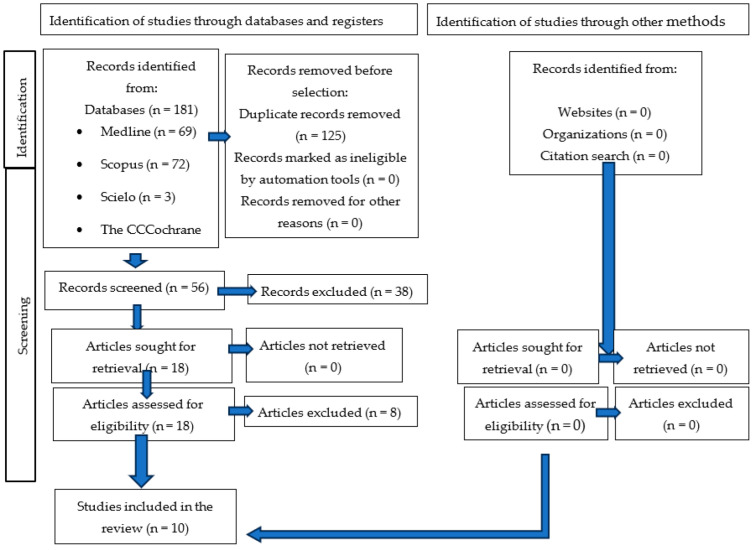
Flow Diagram.

The results of the study selection are shown in Figure 1. Through an exhaustive search of databases, a total of 181 references were found, of which 69 corresponded to Medline PubMed, 72 to SCOPUS, 3 to Scielo, and 37 to The Cochrane Library. Next, 125 duplicate articles were discarded using the bibliographic manager Mendeley, so the title and abstract of 56 articles were analyzed. After an initial screening of 56 articles by title and abstract, 38 were excluded for not meeting the inclusion criteria, such as focusing solely on systemic symptoms of MS or lacking oral health data. The remaining 18 articles were read in full, with an additional 8 studies excluded due to reasons including incomplete outcome reporting (n = 3), a lack of comparison groups (n = 2), duplicate datasets (n = 2), and irrelevance to oral manifestations (n = 1) [20]. The final 10 studies included in this review are those that fulfilled all selection criteria and provided detailed data on oral manifestations in MS patients.

### 3.2. Data Extraction

The outcomes of the data extraction are summarized in Table 2 below. This table presents the key characteristics used to assess the different studies, facilitating a structured comparison across the included research.

The Table 3 presents a summary of the significant associations between various oral signs and symptoms and clinical parameters related to multiple sclerosis, as reported in the included studies. This structured organization allows for a comparative analysis of the findings, facilitates the identification of common trends, and highlights the statistical relevance of each reported association.

### 3.3. Quality Analysis

**Figure 2:**
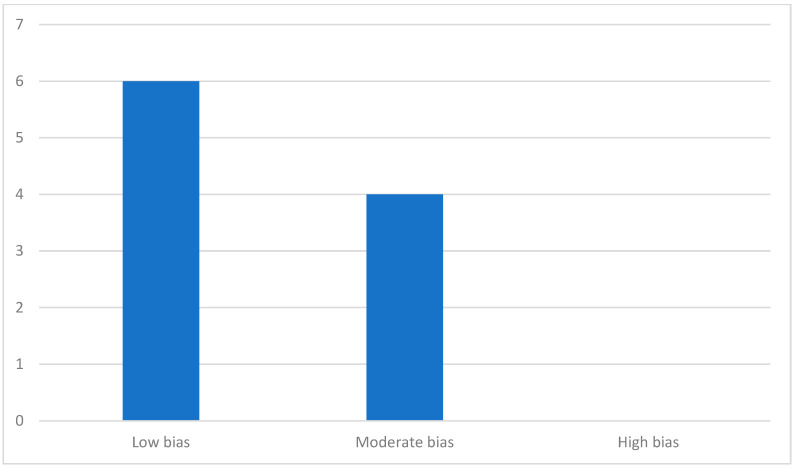
Distribution of studies according to bias.

The results of the quality analysis based on the NOS and JBI guidelines are referenced in Table 4 and Table 5, respectively. According to the analysis, 4 out of the 10 articles were classified as having a moderate risk of bias, while the remaining 6 were considered to have a low risk of bias. None of the studies were evaluated as high risk. A visual summary of the risk of bias distribution is shown in Figure 2.

### 3.4. Bibliometric Analysis

**Figure 3:**
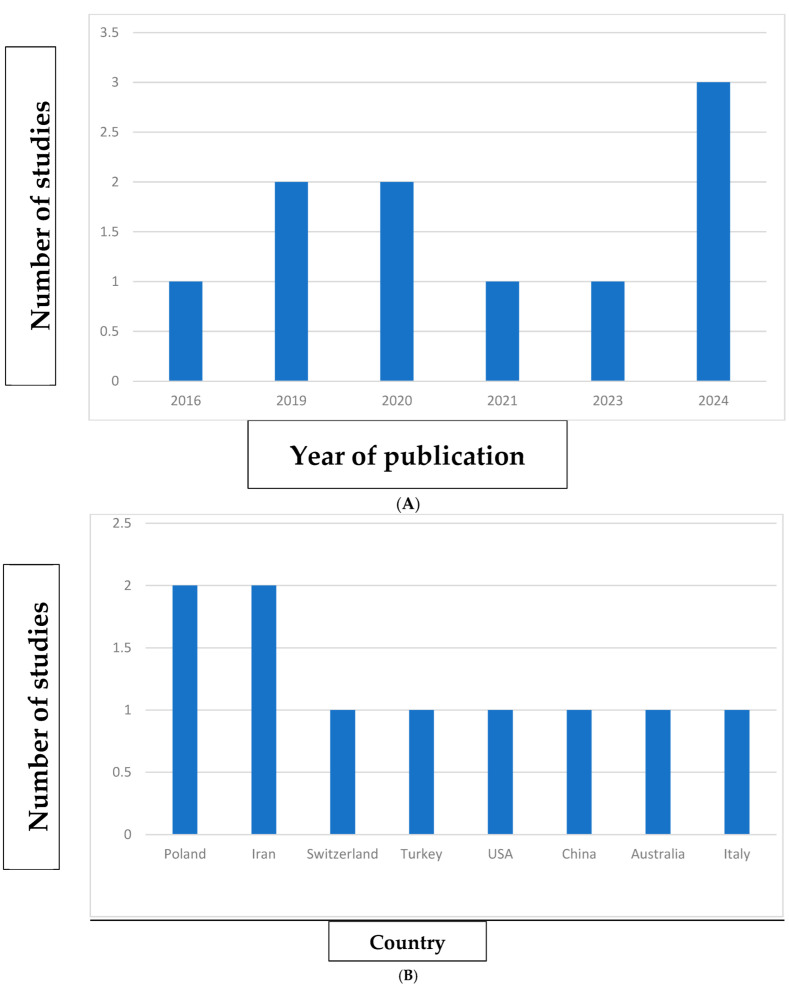

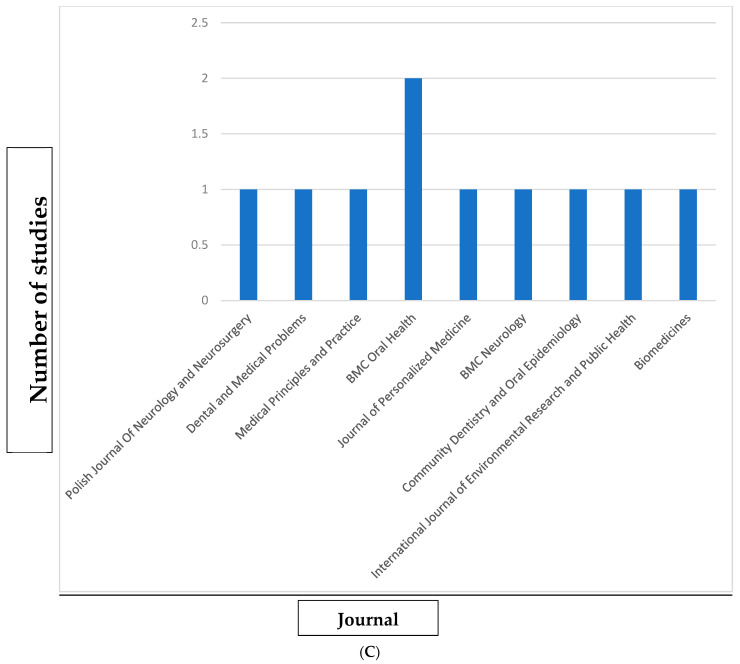
Bibliometric analysis: distribution of included studies by year of publication (**A**); country of publication (**B**); and journal (**C**).

Figure 3 shows the distribution of the articles included in this review according to the year of publication, country of origin, and the journal in which they were published.

With regard to the year of publication, an increase in the number of articles over time can be observed, with a trend toward more recent publications (Figure 3A). No articles were published in the years 2017, 2018, and 2022 but, in contrast, a total of three articles were published in the year 2024. In terms of the country of publication, Poland and Iran were the most represented, each contributing multiple studies, while the remaining countries had only one publication each (Figure 3B). Finally, an analysis of the journals in which the articles appeared reveals a wide range of sources, with ‘BMC Oral Health’ being the most prominent, publishing two of the included studies (Figure 3C).

Regarding the oral manifestations observed, the most commonly reported conditions include dry mouth due to a reduction in salivary flow rate, periodontal involvement characterized by gingival inflammation and increased bleeding index, as well as higher DFMT and plaque index. Additionally, patients often experience tooth pain, dental sensitivity, and alterations in taste perception. These symptoms not only compromise oral health but may also reflect underlying systemic changes, highlighting the importance of comprehensive dental assessment in individuals at risk for neurodegenerative conditions (Table 6).

## 4. Discussion

Multiple sclerosis (MS) is defined as a chronic inflammatory disease of the central nervous system (CNS), characterized by the heterogeneity of its signs and symptoms, as it can affect any part of the CNS [16]. The considerable variability in its clinical manifestations together with the absence of a clear etiology make it difficult to establish an adequate diagnosis and treatment plan.

This review identified hyposalivation, periodontal disease, increased DMFT index, dental sensitivity, and dental pain as the most frequently associated oral manifestations in patients with MS. The data analyzed show that physical disability and disease duration can influence the severity of these oral symptoms, while associations with age and medication use are less consistent. The hypothesis that MS is associated with a higher prevalence of oral manifestations is supported by the findings, although variability in study designs and measures suggests that further research is needed to confirm the strength and direction of these relationships [17,23,27,29].

Several articles establish that dry mouth is the most frequent oral manifestation in patients with multiple sclerosis [17]. Among them, we highlight the study by Labuz-Roszak et al., which identified a significant association between oral dryness and patients with relapsing–remitting multiple sclerosis, as well as those who had received at least one course of steroids in the past. It appears, according to this study, that the MS phenotype developed by the patient influences the incidence or prevalence of decreased salivary flow rate, as it is more common in RRMS patients to a greater extent than in those with PPMS. In addition, this article also reports information about the presence of gingival bleeding, the incidence of which is not significantly associated with the MS phenotype, but with the duration of the disease; it is more prevalent among those who have had the disease for a longer period. In contrast, none of the oral manifestations studied were significantly associated with patient age or the use of disease-modifying drugs. All these results compiled from this cross-sectional study were obtained from the analysis of 199 patients (143 women) with a mean age of 37 years and with a diagnosis of multiple sclerosis (RRMS and PPMS) with a mean duration of the disease of 4 years [17].

Case–control studies, such as the one conducted by Mortazavi et al. [21], also corroborate a significantly lower salivary flow rate in MS patients. In this case, 50 individuals aged between 12 and 54 years were studied, 25 of whom were MS patients forming the case group and the rest were healthy controls. Dental parameters such as the DMFT index for permanent first molars was also evaluated in this article and was non-significantly associated with the presence of the disease and its duration. However, the number of decayed and missing first molars was significantly higher in the case group [21].

On the other hand, it is important to know that the extended disability status scale is a method used to determine the degree of disability in MS patients. In the article published by Hatipoglu et al. [22], the way in which the degree of disability of the patient can influence the development of dental and periodontal impairment is established. A total of 80 patients with multiple sclerosis (64 women and 16 men, aged 18–62 years) were examined, of whom 55 had low physical disability and the rest had high physical disability. The results showed that plaque index, probing depth, and gingival index were higher in patients with a high degree of physical disability, while the number of filled teeth was lower in this group. On the other hand, the results were similar in these two groups regarding the level of clinical attachment and DMFT index. The influence of the degree of physical disability on the presence of oral impairment was not only analyzed in this cross-sectional study, but also in the case–control study by Kiranatl et al. [23], in which a total of 40 patients were examined—20 of them with MS and differentiated into two subgroups according to the EDSS scale, and another 20 healthy patients. While it is true that, overall, the DMFT index was higher and the salivary flow rate lower in MS patients compared to the control group, within the subgroups of MS patients differentiated by EDSS, it was found that the DMFT index was lower and the salivary flow rate higher in patients with a low degree of physical disability compared to patients with a high degree of physical disability. Thus, a statistically significant, negative, and moderate correlation was observed between DMFT index score and salivary flow rate [23].

Regarding periodontal disease, it is known that there are associations between it and systemic diseases such as MS [1,16,22,24,30]. This is because periodontitis operates through biological mechanisms that affect various tissues, leading to inflammatory and functional complications. In this way, the inflammatory adaptation of hematopoietic progenitors in the bone marrow may explain the connection between this oral pathology and chronic inflammatory diseases, with multiple sclerosis being a prominent example. Therefore, considering this relationship, the research investigated how not only periodontitis affects the course of multiple sclerosis, but also how patients with this disease are more susceptible to developing periodontal diseases and how it impacts their progression. This research was analyzed in the study published by Chatzopoulos et al. [24], where it was concluded, after examining 2807 records, that multiple sclerosis is significantly associated with the rate of progression of periodontitis, with MS patients presenting a higher degree of periodontitis. However, this systemic impact does not significantly influence the stage of periodontal disease [24].

In the study by Wu et al. [26], further investigation into this bidirectional relationship was conducted through a bioinformatics analysis, which found that the genes FAM46C, CFI, and DDIT4L are common to both pathologies (periodontitis and multiple sclerosis), and could be used as diagnostic biomarkers. It was also demonstrated that immune responses triggered by B and T lymphocytes are important in the pathogenesis of both periodontitis and MS, and that both conditions are associated with an elevated presence of Porphyromonas gingivalis [26].

Another relevant oral manifestation that appears in patients with MS is the reduced dental sensitivity. To analyze this topic, the study published by Owlia et al. [25] will be used, in which a total of 69 patients were examined, 34 of whom had RRMS, and the rest were healthy individuals. To avoid bias, both groups had similar ages and gender proportions. The assessment of the degree of dental sensitivity was performed using an electrical pulp test on the maxillary central incisors of everyone in the sample, as they are easier to isolate and access and have fewer cavities and connections with adjacent teeth. The pulp response to this test was significantly different between the two groups, with a lower response observed in MS patients. However, it was shown that the duration of the disease was not significantly related to the threshold for electrical dental stimulation [25].

In the articles published by Sexton et al. [27], Covello et al. [28], and Kapel-Regula et al. [29], other oral signs and symptoms such as dental pain or the number of lost teeth are correlated with multiple sclerosis. In the first two, which are cross-sectional studies, questionnaires were used to determine the prevalence of oral manifestations. In the Sexton et al. study, 1523 people were surveyed, and it was found that the presence of dental pain is significantly more likely to occur in MS patients, although it is not associated with the course of the disease [27]. A similar result was obtained in the article by Covello et al., which also determined that the highest prevalence of dental pain, along with the presence of gingival inflammation and xerostomia, negatively affects the individual’s quality of life [28].

Finally, in the case–control study published by Kapel-Regula et al. [29], in which a total of 152 people were examined, 101 of whom had RRMS and were treated with four different types of treatments, while the rest were healthy individuals, a significant association was established between dry mouth and MS. The duration of the disease did not show a positive correlation with this oral symptom, but it was correlated with the number of lost teeth, as well as the expanded disability status scale. In addition, RRMS was significantly associated with a higher plaque index and a lower gingival bleeding index [29].

Like the other published articles, this review also has limitations. The fact that a high number of different articles with more detailed and elaborate information on oral manifestations in the databases used was not found represents a significant limitation. Moreover, only observational studies published in English or Spanish were included, which further reduced the number of articles included in this review.

## 5. Conclusions

This systematic review indicates that individuals with multiple sclerosis commonly exhibit oral manifestations such as hyposalivation, increased periodontal disease prevalence, higher DMFT scores, and orofacial pain. These findings suggest a bidirectional relationship between neurological impairment and oral health status. Given the variability in study design and quality, further standardized research is necessary to confirm these associations and to develop targeted oral health strategies for patients with MS.

## Figures and Tables

**Table 1:** Search strategy.

Databases	Search Field	Results
Medline (PubMed)	1# “multiple sclerosis” OR “multiple sclerosis, relapsing-remitting” OR “multiple sclerosis, progressive” OR “multiple sclerosis, chronic progressive” OR “multiple sclerosis, primary progressive” OR “multiple sclerosis, secondary progressive” OR “multiple sclerosis, clinical remission” OR “demyelinating disease”	51.513
	2# “oral manifestations” OR “manifestation, oral” OR “manifestations, oral” OR “oral manifestation” OR “oral lesions” OR “oral ulcers” OR “mucosal lesions” OR “oral health”	55.175
	**1# AND 2#**	69
SCOPUS	1# “multiple sclerosis” OR “multiple sclerosis, relapsing-remitting” OR “multiple sclerosis, progressive” OR “multiple sclerosis, chronic progressive” OR “multiple sclerosis, primary progressive” OR “multiple sclerosis, secondary progressive” OR “multiple sclerosis, clinical remission” OR “demyelinating disease”	4
	2# “oral manifestations” OR “manifestation, oral” OR “manifestations, oral” OR “oral manifestation” OR “oral lesions” OR “oral ulcers” OR “mucosal lesions” OR “oral health”	1.913
	**1# AND 2#**	72
Scielo	1# “multiple sclerosis” OR “multiple sclerosis, relapsing-remitting” OR “multiple sclerosis, progressive” OR “multiple sclerosis, chronic progressive” OR “multiple sclerosis, primary progressive” OR “multiple sclerosis, secondary progressive” OR “multiple sclerosis, clinical remission” OR “demyelinating disease”	843
	2# “oral manifestations” OR “manifestation, oral” OR “manifestations, oral” OR “oral manifestation” OR “oral lesions” OR “oral ulcers” OR “mucosal lesions” OR “oral health”	256
	**1# AND 2#**	3
The Cochrane Library	1# “multiple sclerosis” OR “multiple sclerosis, relapsing-remitting” OR “multiple sclerosis, progressive” OR “multiple sclerosis, chronic progressive” OR “multiple sclerosis, primary progressive” OR “multiple sclerosis, secondary progressive” OR “multiple sclerosis, clinical remission” OR “demyelinating disease”	93
	2# “oral manifestations” OR “manifestation, oral” OR “manifestations, oral” OR “oral manifestation” OR “oral lesions” OR “oral ulcers” OR “mucosal lesions” OR “oral health”	29
	**1# AND 2#**	37

**Table 2:** A description of the differentiated variables for each of the articles examined.

Author	Year	Study Type	Most Prevalent Oral Manifestations	Treatment	Differential Diagnosis	Associated Conditions
Labuz-Roszak et al. [17]	2019	Cross-sectional study	Dry mouth and gingival bleeding	Education and awareness about oral health	-	Development of MS and presence of gingival bleeding and dry mouth
Mortazavi et al. [21]	2020	Case–control study	Reduced salivary flow and higher DMFT (decayed, missing, and filled teeth) index	-	-	Calcium and phosphorus levels and patients with MS
Hatipoglu et al. [22]	2016	Cross-sectional study	Higher periodontal indices	-	-	Plaque index, probing depth, clinical attachment level, gingival index, and DMFT index in MS patients. Also includes association with the expanded disability status scale
Kiranatl et al. [23].	2024	Case–control study	Decreased salivary flow and higher DMFT index	-	-	Salivary flow rate and DMFT index in RRMS patients
Chatzopoulos et al. [24]	2023	Cross-sectional study	Periodontal disease	-	-	Degree, progression, and stage of periodontal disease in MS patients
Owlia et al. [25]	2021	Case–control study	Lower dental pulp sensitivity	-	-	Response to electric pulp sensitivity test, MS patients, and disease duration
Wu et al. [26]	2024	Case–control study	Periodontitis	-	-	T and B cells significantly influence MS development and periodontitis. CFI, DDIT4L, and FAM46C as potential biomarkers in periodontitis and MS
Sexton et al. [27]	2018	Cross-sectional study	Dry mouth, dental sensitivity, taste alteration, and orofacial pain	-	Among tooth pain, dental sensitivity, and orofacial pain	Toothache and clinical course of MS. Dry mouth and sensitivity in MS patients
Covello et al. [28]	2020	Cross-sectional study	Gingival inflammation, xerostomia, dysphagia, and neuralgia	Instruction in the use of oral hygiene devices	Between trigeminal neuralgia and pain caused by pressure or chewing forces	Prevalence of gingival bleeding, dental sensitivity, and tooth pain related to the gender of MS patients
Kapel-Regula et al. [29]	2024	Case–control study	Higher plaque index, lost teeth, dry mouth, and taste alterations	Ongoing care and prevention (fluoride toothpaste and saliva substitutes). Oral hygiene education	-	Relationship between lost teeth, filling types, plaque index, gingival bleeding, dry mouth in MS patients, disability degree, and disease duration

**Table 3:** Oral signs and symptoms and their significant association.

Author	Parameter 1	Parameter 2	Significance Level
Labuz-Roszak et al. [17]	1. Dry mouth and gingival bleeding	1. MS phenotype 2. Disease duration 3. Age 4. Use or not of disease-modifying drugs	1. Significant *p* = 0.023 (more frequent in SPMS, regarding dry mouth) 2. Significant *p* = 0.002 3. Not significant *p* = 0.392 and *p* = 0.877 4. Not significant *p* = 0.994 and *p* = 0.662
Mortazavi et al. [21]	1. Reduced salivary flow rate 2. Salivary calcium and phosphorus levels 3. DMFT index 4. Number of decayed and missing permanent first molars	1. MS patients 2. MS patients 3. In first permanent molars in MS patients 4. In MS patients	1. Significant *p* = 0.001 2. Significant *p* = 0.011 and *p* = 0.020 3. Not significant *p* = 0.452 4. Significant *p* = 0.038 and *p* = 0.019 respectively
Hatipoglu et al. [22]	1. Plaque index, probing depth, gingival index, and number of restored teeth 2. Number of decayed teeth 3. Clinical attachment level, DMFT index (decayed, missing, and filled teeth)	1. Disability level in MS patients 2. Expanded disability status scale in MS patients with high physical disability 3. Subgroups of MS patients according to disability degree	1. Significant *p* < 0.05 2. Significant *p* = 0.005 3. Similar
Kiranatl et al. [23]	1. DMFT index 2. Salivary flow rate 3. DMFT index and salivary flow rate	1. MS patients compared to control group 2. MS patients compared to control group 3. Disability score on expanded disability status scale	1. Significant *p* = 0.004 2. Significant *p* = 0.002 3. Not significant *p* = 0.339 and significant *p* = 0.002 respectively
Chatzopoulos et al. [24]	1. Periodontal disease progression rate 2. Degree of periodontal disease 3. Stage of periodontal disease	1. MS 2. MS 3. MS	1. Significant *p* < 0.001 2. Significant *p* < 0.05 3. Not significant *p* > 0.99
Owlia et al. [25]	1. Response of teeth to electric pulp sensitivity test 2. Electrical stimulation threshold of dental pulp	1. MS patients compared to control group 2. MS duration	1. Significant *p* = 0.0001 2. Not significant *p* = 0.78
Wu et al. [26]	1. Expression of FAM46C, CFI, and DDIT4L biomarkers	1. Development of periodontal disease and multiple sclerosis	1. Significant *p* < 0.05
Sexton et al. [27]	1. Tooth pain, dry mouth, and dental sensitivity 2. Tooth pain	1. People with MS 2. Clinical course of multiple sclerosis	1. Significant *p* < 0.05 2. Not significant *p* > 0.05
Covello et al. [28]	1. Tooth pain, dental sensitivity, and gingival bleeding	1. Gender of MS patients	1. Not significant *p* > 0.05
Kapel-Regula et al. [29]	1. Higher number of lost teeth, amalgam fillings, dry mouth, and elevated plaque index 2. Fewer composite fillings and lower gingival bleeding index 3. Lost teeth 4. Lower gingival bleeding index	1. RRMS patients 2. RRMS patients 3. Disease duration and expanded disability status scale score 4. Expanded disability status scale	1. Significant *p* < 0.05 2. Significant *p* < 0.05 3. Significant *p* < 0.05 4. Significant *p* < 0.05

**Table 4:** The quality assessment of the studies using the adapted version of NOS for case–control studies.

Case–Control Studies (NOS)	Selection	Comparability	Exposure	Total Score
Mortazavi et al. [21]	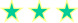	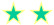	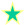	6
Kiranatl et al. [23]	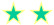	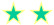	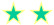	5
Owlia et al. [25]	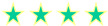	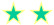	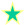	7
Wu et al. [26]	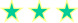	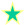	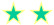	6
Kapel-Regula et al. [29]	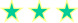	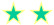	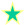	6

**Table 5:** JBI checklist evaluation.

Article Title	Clear Inclusion Criteria	Subjects and Setting Described	Exposure Measured Validly	Standard Criteria for Condition	Confounding Factors Identified	Strategies to Deal with Confounding	Outcomes Measured Validly	Appropriate Statistical Analysis	Overall Appraisal	%
Sexton et al. (2019) [27]	Yes	Yes	Yes	Yes	Yes	Yes	Yes	Yes	Include	100
Hatipoglu et al. (2016) [22]	Yes	Yes	Yes	Yes	Yes	Partial	Yes	Yes	Include	87.5
Chatzopoulos et al. (2023) [24]	Yes	Yes	Yes	Yes	Yes	Yes	Yes	Yes	Include	100
Covello et al. (2020) [28]	Yes	Yes	Yes	Yes	Partial	Partial	Yes	Yes	Include	75
Labuz-Osrek et al. (2019) [17]	Yes	Yes	Yes	Yes	Yes	Yes	Yes	Yes	Include	100

**Table 6:** Most prevalent oral microbiota in multiple sclerosis.

Oral Manifestations
- Dry mouth (reduction in salivary flow rate) - Periodontal involvement (periodontal disease, bleeding index, gingival inflammation) - Higher DFMT and plaque index - Tooth pain - Dental sensitivity - Taste alterations
